# Reference gene and small RNA data from multiple tissues of *Davidia involucrata* Baill

**DOI:** 10.1038/s41597-019-0190-7

**Published:** 2019-09-24

**Authors:** Hua Yang, Chengran Zhou, Guolin Li, Jing Wang, Ping Gao, Maolin Wang, Rui Wang, Yun Zhao

**Affiliations:** Key Laboratory of Bio-Resource and Eco-Environment of Ministry of Education, College of Life Sciences, Sichuan University, Chengdu, 610065 China

**Keywords:** Data publication and archiving, Plant evolution

## Abstract

*Davidia involucrata* Baill. is a rare plant endemic to China. Its exclusive evolutionary position and specific floral organs endow it with a high research value. However, a lack of genomic resources has constrained the study of *D. involucrata* functional genomics. Here, we report *D. involucrata* transcriptome reads from different floral tissues pooled from six individuals at two developmental stages using Illumina HiSeq technology and the construction of a high-quality reference gene set containing a total of 104,463 unigenes with an N50 of 1,693 bp and 48,529 high-quality coding sequences. The transcriptome data exhibited 89.24% full-length completeness with respect to the benchmarking universal single-copy (BUSCO) dataset and a PLAZA CoreGF weighted score of 98.85%. In total, 65,534 (62.73%) unigenes were functionally annotated, including 58 transcription factor families and 44,327 simple sequence repeats (SSRs). In addition, 96 known and 112 novel miRNAs were identified in the parallel small RNA sequencing of each sample. All these high-quality data could provide a valuable annotated gene set for subsequent studies of *D. involucrata*.

## Abstract (summary)

**Table:** 

Measurement(s)	small RNA sequencing assay • transcription profiling assay
Technology Type(s)	RNA sequencing
Factor Type(s)	developmental stage
Sample Characteristic - Organism	Davidia involucrata

Machine-accessible metadata file describing the reported data: 10.6084/m9.figshare.9772118

## Background & Summary

*Davidia involucrata* Baill., also called dove tree or handkerchief tree, is the sole species in the genus Davidia (Davidiaceae^1^ or Nyssaceae^2^) and is listed as a “first-grade” nationally protected plant in China^3,4^. It is a Tertiary paleotropical relic plant species that is rare in China and usually regarded as a “botanic living fossil”^5^. Its distribution is limited to the subtropical mountains of central to southwestern China; natural populations are often found in deciduous or evergreen broad-leaf forests at elevations of 1100–2600 m^6^. *D. involucrata* is not only an endangered and rare relic species but also famous as an ornamental plant by virtue of the pair of large white bracts that surround the small flowers and create the appearance of doves perching among its branches, giving the tree its common name^7^. The most unusual characteristics of *D. involucrata* are its floral organs, of which the inflorescences contain either a mixture of hermaphrodite and many male flowers or entirely male flowers; the flowers are without petals but have large, unequal, paired paper-like bracts instead. These intriguing bracts originally appear small and green, resembling leaves, but they increase in size and turn white as the flowers mature, and then, finally, turn to brown and yellow before being shed^8,9^. Bracts are special organs that appear in the reproductive development of plants, and *D. involucrata* is undoubtedly an ideal subject for the study of bracts and the developmental mechanisms of specific flower organs.

To date, studies of *D. involucrata* have mainly focused on the macroscopic and phenotypic levels, such as taxonomy, morphology, physiology, ecology, reproduction and so on^9–13^. However, research at the molecular level continues to progress slowly, which could result from the distribution of this species, which is intermittently scattered throughout southwest China, and its growth characteristics; *D. involucrata* is slow-growing, occurs in harsh growth conditions and has a low survival rate. Therefore, deeper research at the molecular level is critical for this endangered plant.

Studies of the molecular mechanisms underlying the growth and development of *D. involucrata* as well as its unique bracts and floral organs could further reveal the evolution of floral development, which remains unclear due to a lack of high-throughput data. To date, many molecular studies have focused on exploring and analysing traditional markers, such as simple sequence repeats (SSRs)^14–16^, random amplified polymorphic DNA (RAPD) markers^17^, microsatellites^18^ and chloroplast genes^19^, or utilized functional genes or factors, such as a MYB transcription factor from *D. involucrata* (*DiMYB1)*^20^, a cold-induced gene (*DiRCI*)^21^, a clathrin adaptor complex gene (*DiCAC)*^22^ and so on. At the same time, the transcriptome of the seed^23^ and the chloroplast genome^24^ have been reported. However, transcriptomic and sRNA data from some crucial tissues, for example, the floral organs and, specifically, the bracts, have not been examined in depth^23–25^.

Here, we established a complete gene set from multiple tissues of *D. involucrata* by means of next generation sequencing technology. This gene set would be useful for further studies. For example, it could be used as a reference for gene characterization, such as expression analysis, gene cloning and phylogenetic analysis, and it could also be used for gene model annotation once the genome is sequenced. In addition, the transcriptome annotations could be used to explore the crucial genes in flower development and bract development, which are of great significance to reveal the evolution of the floral organs in angiosperms. Furthermore, the small RNAs of each sample were sequenced and annotated in parallel to provide more information about sRNA-related regulatory mechanisms during floral organ development. The whole transcriptomes established herein lay a foundation for plant molecular marker-assisted breeding, evolutionary and developmental analysis, and even plant protection in the future.

## Methods

### Sample collection

**Fig. 1:**
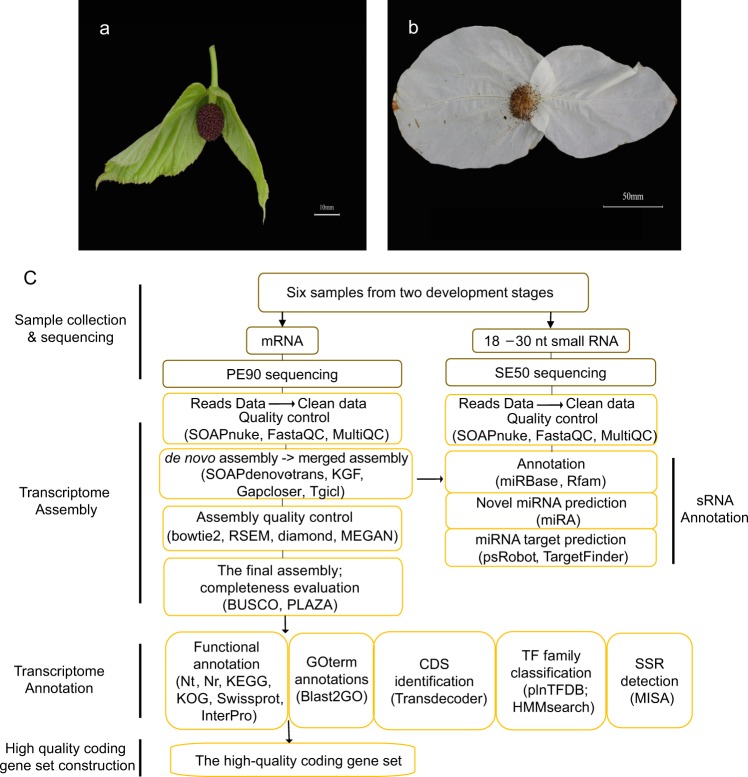
Schematic pipeline illustrating analysis of the whole transcriptome dataset. (**a**) The young inflorescences of *D. involucrata*. (**b**) The mature inflorescences of *D. involucrata*. (**c**) After library construction, mRNAs were sequenced with a PE90 strategy, and sRNAs were sequenced with an SE50 strategy. After low-quality read removal, the transcriptome of each sample was assembled using SOAPdenovo-trans, KGF and GapCloser. After clustering using TGICL, the final assemblies were obtained, and addition evaluation and annotation were performed.

The floral organ samples were collected from 6 blooming individuals at two developmental stages (3 individuals for each stage) in a wild population in the county town of Yingjing, Yaan, Sichuan Province, in April 2014 (Fig. 1a,b). Floral organ growth stages were defined based on the colour changes of the bracts. One stage was young inflorescences with small, green bracts (expanded to approximately <8 cm in length) resembling leaves, and the dark purple anthers were immature (Fig. 1a). The other stage was mature inflorescences with large white bracts (expanded to approximately >15 cm in length), and the anthers were mature (Fig. 1b). Each tissue sample collected from each plant was approximately thumbnail-sized and was immediately stored in RNA Fixer solution (Bioteke, China) for further use.

The floral organ samples (Table 1) were named YB (young bracts: a pool of similar samples collected from the growing green bracts of three individuals), LB (mature bracts: a pool of similar samples collected from the mature white bracts of three individuals), LX (mature stamens: a pool of similar samples containing the complete, mature stamens from three individuals), LC (mature pistils: a pool of similar samples containing the complete, mature pistils from three individuals), YR (young mixed samples: a pool containing flowers at the early blooming stage including equal amounts of complete stamens and complete pistils from three individuals), ZR (mixed samples: a pool of all collected floral organ samples with added leaves, shoots, and roots at two periods from all six individuals).

**Table 1:** Summary of transcriptome reads.

Sample name	Sample accession ID	Clean PE reads	Read length (bp)	Clean bases	Q20	GC content	Total mapped reads	Total mapped percentage	SRA accession ID
ZR	SAMN10721419	22,384,892	2 × 90	4,029,280,560	99.18%	44.69%	40,721,814	90.96%	SRR8427005
YB	SAMN10721417	22,363,088	2 × 90	4,025,355,840	99.18%	44.57%	40,511,938	90.58%	SRR8427013
YR	SAMN10721418	22,439,409	2 × 90	4,039,093,620	99.18%	44.62%	40,982,224	91.32%	SRR8427004
LB	SAMN10721416	22,350,513	2 × 90	4,023,092,340	99.19%	44.91%	40,725,710	91.11%	SRR8427010
LC	SAMN10721415	22,216,991	2 × 90	3,999,058,380	99.17%	44.90%	39,992,240	90.00%	SRR8427011
LX	SAMN10721414	22,388,190	2 × 90	4,029,874,200	98.86%	44.94%	40,853,690	91.24%	SRR8427012

### Total RNA extraction

Total RNA extraction was performed using the TRIzol Reagent (Invitrogen, Carlsbad, CA) according to the manufacturer’s instructions, followed by quality assessment on an Agilent 2100.

### Transcriptome sequencing and filtering

The mRNA was extracted from the total RNA with oligo (dT)-attached magnetic beads, and a cDNA library with an insert size of 250 bp was constructed using the TruSeq RNA Sample Preparation Kit according to the manufacturer’s protocol (Illumina Inc., San Diego, CA). Library generation yielding 2 × 90 bp paired-end reads and sequencing (Illumina HiSeq 2000) were performed at BGI-Shenzhen. Low-quality reads matching one or more of the following criteria were filtered out using SOAPnuke (v1.5.6)^26^: reads containing adaptor contamination; reads including more than 5% of the unknown base “N”; reads including more than 20% bases with quality values lower than 10.

### sRNA sequencing and filtering

RNA segments of different sizes from 18–30 nt were separated from the total RNA by 15% denaturing PAGE and selected for small RNA library construction using the Illumina TruSeq Small RNA Sample Preparation Kit according to the manufacturer’s protocols (Illumina Inc.). Library generation yielding 50 bp single-end reads and sequencing (Illumina HiSeq. 2000) were also performed at BGI-Shenzhen. The contaminant tags and low-quality tags were removed using SOAPnuke (v1.5.6)^26^: (1) tags with 5′ primer contaminants, oversized insertion, poly-A; (2) tags without 3′ primer or insert tags; (3) tags shorter than 18 nt. After the removal of low-quality reads, clean reads (Table 2) were retained and used in subsequent analyses (Fig. 1). The quality of all clean reads was assessed with FastQC (http://www.bioinformatics.bbsrc.ac.uk/projects/fastqc) and MultiQC (v1.8)^27^.

**Table 2:** Summary of sRNA reads.

Sample name	Clean reads	Clean bases	Read length (bp)	Q20	GC content	SRA Accession ID
ZR	11,019,818	250,536,916	49	99.01%	46.78%	SRR8427007
YB	11,025,370	251,011,999	49	99.17%	46.53%	SRR8427015
YR	11,063,468	256,105,784	49	99.17%	45.81%	SRR8427006
LB	11,021,444	252,095,057	49	99.04%	46.72%	SRR8427008
LC	11,085,430	258,116,171	49	99.19%	45.72%	SRR8427009
LX	11,063,531	251,807,174	49	99.01%	46.72%	SRR8427014

### Transcriptome assembly

Transcriptome assembly was completed using a combined assembly strategy. Briefly, the RNA-Seq reads were assembled using SOAPdenovo-Trans (version 1.01)^28^ with the following settings: “-K 31-i 20 -e 2 -M 3-L 100”. The gaps were filled using KGF (v1.19) and the GapCloser tool (v1.12)^29^. All assemblies were merged into one large dataset using TGICL (v2.0.6)^30^ with the parameters “-l 40 -c 10 -v 25 -O ‘-repeat_stringency 0.95 -minmatch 35 -minscore 35’”.

To remove unreliably assembled transcripts, clean reads were aligned to the assembly using Bowtie2^31^ with the parameters “–sensitive–score-min L,0,-0.1 -I 1 -X 1000–mp 1,1–np 1–no-mixed–no-discordant”, and the fragments per kilobase of exon model per million reads mapped (FPKM) values of the transcripts were calculated using the tool rsem-calculate-expression in RSEM (v1.2.21)^32^. The sequences with FPKM values of zero were removed from the assembly. Then, the lowest common ancestor (LCA) algorithm was applied to filter out contaminants. First, the assembly was used to search against the NCBI non-redundant protein database (Nr) using Diamond with e-value < 1e-5. The tool Blast2lca in MEGAN (v6.15.2)^33^ with the parameter “–minScore 75” was used to apply the LCA alignment and produce taxonomic classifications. Non-Viridiplantae sequences were removed according to their classifications. All unreliably unigenes of each individual were also filtered. Last, we obtained the final transcriptome assembly (Table 3**)**.

**Table 3:** Summary of unigenes and the final assembly.

Type	Sample	Total number	Total length	N50	GC content	TSA Accession ID
Unigenes	LB	57,173	49,713,731	1416	41.95%	GHEJ00000000
	LC	58,948	51,711,196	1,424	42.18%	GHEO00000000
	LX	54,395	45,213,861	1,343	42.27%	GHEP00000000
	YB	56,896	49,360,048	1,379	42.01%	GHEQ00000000
	YR	60,705	50,204,010	1,341	41.85%	GHET00000000
	ZR	63,510	54,067,004	1,368	42.15%	GHER00000000
The final assembly	All	104,463	109,238,123	1,693	41.58%	GHES00000000

## Transcriptome Annotation

### Functional annotation

Functional annotations were performed using a sequence-based search method (Table 4). First, the final assembly was annotated by searching against the NCBI non-redundant nucleotide (Nt) database^34^ using BLASTn (v2.4.0)^35^ with e-value < 1e-5, Viridiplantae-related non-redundant proteins in the Nr database, Swiss-Prot in UniProtKB^36^, the Kyoto Encyclopedia of Genes and Genomes (KEGG) pathway database^37^ and clusters of euKaryotic Orthologous Groups (KOG)^38^ using BLASTx (v2.4.0) with e-value < 1e-5, and the InterPro database^39^ using InterProScan (v5)^40^ with the default parameters^36^. Gene Ontology (GO)^41^ annotation was performed using Blast2GO (v2.5.0)^42^ based on the Nr annotation results.

**Table 4:** Summary of functional annotation.

Values	Total	Nr	Nt	Swissprot	KEGG	KOG	Interpro	GO	Overall
Number	104,463	60,535	51,601	40,664	44,639	46,019	51,446	31,080	65,534
Percentage	100%	57.95%	49.40%	38.93%	42.73%	44.05%	49.25%	29.75%	62.73%

### Identification of coding gene set

First, the open reading frames (ORFs) of each sequence were predicted using TransDecoder (v3.0.1) as implemented in Trinity^43^. Then, the ORFs were searched against Swiss-Prot^36^ using Diamond Blastp (v0.8.31)^44^, and the output file was searched against Pfam^45^ using Hmmscan (v3)^46^. Finally, CDSs were predicted using TransDecoder with the results of the previous step (Table 5).

**Table 5:** Summary of CDS prediction and coding gene set.

Type	Total number	Total length	N50	N90	Max length	Min length	Sequence GC content
Predicted CDS by transdecoder	58,561	60,633,393	1,359	489	12,852	297	44.99%
The final high-quality coding gene set	48,529	55,792,500	1,437	591	12,852	297	44.98%

### Identification of high-quality annotated coding gene set

To identify high-quality coding genes, we used the following pipeline. (1) All transcripts had matches with protein databases, and the best hit for each transcript (in the following order: Nr, Swiss-Prot, KEGG, KOG and InterPro) was extracted. (2) The annotated coding region and functional annotation for each transcript were selected from the best hit. (3) The transcript with the longest CDS was chosen if its annotated coding region could also be identified by TransDecoder. (4) After filtering out sequences with more than 5 Ns in the last 10 bases or more than 15 Ns in the last 50 bases, the eligible CDS of each transcript was extracted as the final coding gene. In total, 51,247 annotated coding genes with a minimal length of 297 nt and a maximal length of 12,852 nt were identified as high-quality coding genes (Table 5).

### Transcription factor family classification

**Fig. 2:**
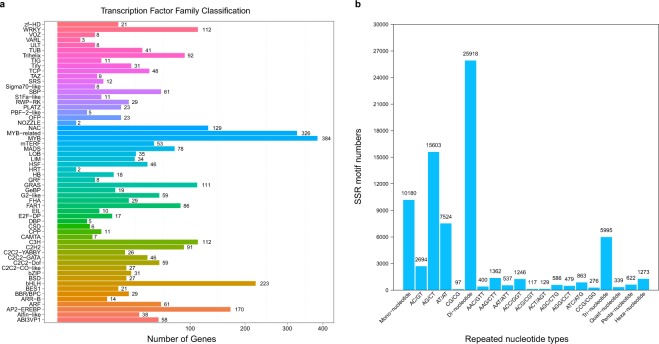
Transcription factor family and simple sequence repeat statistics. (**a**) Transcription factor family classifications of the assembled sequences. (**b**) SSR statistics.

The open reading frame of each sequence was classified using getorf (EMBOSS:6.5.7.0)^47^, and the transcriptome factor (TF) family was identified based on plant TF domains in the plant TF database (PlnTFDB)^48^ using Hmmsearch^46^. In total, 3,064 genes were arranged in 58 TF families, and the top three annotated families were the Myb DNA-binding domain families MYB and MYB-related and the basic helix-loop-helix (bHLH) family (Fig. 2a).

### Simple sequence repeat detection

SSRs in the transcriptome sequences were detected using MISA (v1.043)^49^ with the definition parameters “1-12,2-6,3-5,4-5,5-4,6-4” and an interruption parameter of “100”. The total number of identified SSRs was 44,327, and the number of SSR-containing sequences was 20,739 (Fig. 2b).

## sRNA Annotation

### Small RNA annotation

Because of the lack of a *D. involucrata* special sRNA reference dataset, we used the microRNA datasets of *Arabidopsis lyrata* from miRBase (release 22)^50^ and RNA datasets from the Rfam database (v12.1)^51^ as the known small RNA reference databases. Clean reads were mapped to the miRBase database using Bowtie2 (v2.2.5)^31^ with “–sensitive -L 16” and the Rfam database using cmsearch in INFERNAL (v1.1.2)^52^ with “–noali” to obtain the annotations. Aligned tags were annotated after filtering reads with more than one mismatch out of each alignment. A total of 96 known miRNAs were annotated in this study.

### Novel miRNA prediction

**Fig. 3:**
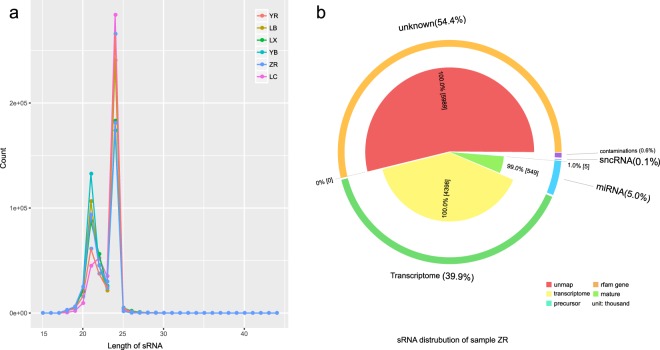
Length distributions of sRNA samples and sRNA annotation distribution of sample ZR. (**a**) Length distributions of sRNA. The peak of each sample was located at 24 nt. (**b**) sRNA annotation distribution of sample ZR.

Because no more than 10% of the small RNA tags were annotated, miRA software^53^ was applied to predict novel miRNAs. First, the clean reads of each sample were mapped to the “genome” reference (the merged transcriptome) using Bowtie2 with the parameters “-L 16–rdg 1,10–rfg 1,10”. After removing the unreliable transcriptome sequences from the alignments, 45.28%, 44.08%, 43.62%, 44.21%, 38.90% and 54.74% tags could be mapped to the transcriptome for ZR, YR, YB, LB, LC and LX, respectively. After filtering out the annotated sRNA reads, the aligned read tags from all samples were combined into one FASTA file. Then, the combined FASTA file was mapped to the genome reference using Bowtie2 with “-f -l 16”, and the alignment file was filtered using SAMtools (v1.3.1)^54^ with “view -hS -F 4”. Finally, miRA (v1.2.0) with the default parameters was used to predict novel miRNAs. In total, 112 novel miRNAs were identified. The annotation and prediction results are summarized in Table 6 **(**Table 6, Fig. 3**)**.

**Table 6:** sRNA annotation and prediction results.

Sample Type	ZR		YR		YB		LB		LC		LX	
	Count	Percentage	Count	Percentage	Count	Percentage	Count	Percentage	Count	Percentage	Count	Percentage
Mature miRNA	549995	4.99%	594518	5.38%	4315731	39.41%	714260	6.48%	90223	0.81%	962419	8.70%
Precursor miRNA	5004	0.05%	2835	0.03%	5444	0.05%	5954	0.05%	1404	0.01%	5033	0.05%
Rfam other sncRNA	554	0.01%	580	0.01%	565	0.01%	572	0.01%	418	0	1321	0.01%
rRNA	5725	0.05%	16442	0.15%	6536	0.06%	14169	0.13%	8599	0.08%	66842	0.60%
snoRNA	436	0	512	0	292	0	316	0	178	0	817	0.01%
snRNA	1395	0.01%	869	0.01%	927	0.01%	1686	0.02%	838	0.01%	1459	0.01%
tRNA	7	0	51	0	10	0	20	0	1	0	16	0
Transcriptome	4398410	39.91%	4233656	38.31%	4379692	39.15%	4106787	37.05%	4187990	37.78%	4979574	45.64%
Unmap sRNA	5989783	54.35%	6144853	55.89%	6178346	56.05%	6111352	55.47%	6741014	60.82%	4972517	44.97%
Mapped onto unreliable transcripts	68509	0.62%	57680	0.52%	71934	0.65%	72354	0.65%	62938	0.57%	74092	0.67%
Total	11019818	100%	11051996	100%	11022568	100%	11018338	100%	11084396	100%	11057255	100%

### miRNA target prediction

psRobot (v1.2)^55^ and TargetFinder (v1.0)^56^ with the default parameters were used to predict miRNA targets. The intersection of the target genes was extracted as the final prediction target result. The final intersection results included 172 miRNAs and 1,737 target genes.

## Data Records

The final transcriptome and related data are published under the International Nucleotide Sequence Database Collaboration BioProject PRJNA513477 (https://identifiers.org/ncbi/bioproject:PRJNA513477) and CNGB Nucleotide Sequence Archive (CNSA) project CNP0000260 (https://db.cngb.org/search/project/CNP0000260). The read files were deposited in the NCBI Sequence Read Archive^57^. The final high-quality coding gene set and the transcriptome assemblies were deposited in NCBI Transcriptome Shotgun Assembly^58^ and CNSA. Annotation data set was uploaded to figshare^59^.

## Technical Validation

### Quality control and data statistics

**Fig. 4:**
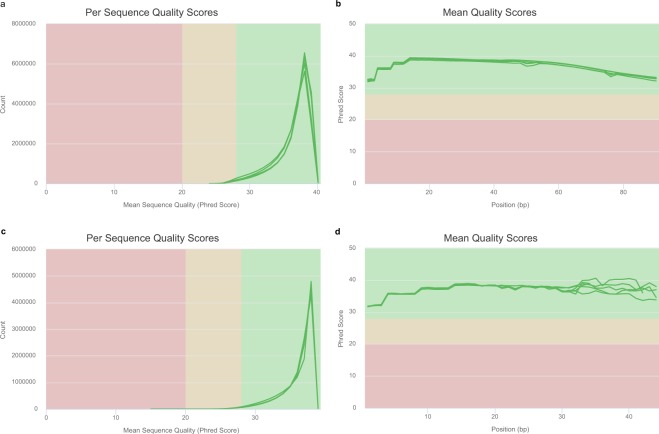
Quality assessment. Read count distributions by mean sequence quality of transcriptome reads (**a**) and sRNA reads (**c**). Mean quality score distributions of transcriptome reads (**b**) and sRNA reads (**d**).

To control the sequencing quality, the counts of clean reads, total bases, quality scores and GC content were calculated for each sample using FastQC and MultiQC (Tables 1, 2 and Fig. 4). The mean read counts per quality scores and the mean quality scores in each base position were higher than 30. The length distribution of clean small RNA tags showed that the peak of each sample was located at 24 nt (Fig. 3).

### Assembly quality control

Different tissues from different trees were collected in this study to comprehensively cover the *D. involucrata* transcriptome. Because reads were aligned to the merged assembly, we summarized the mapping percentages in Table 1 and then calculated the FPKM value for each sequence. The read mapping results showed that more than 90% of the reads were mapped to the transcriptome, and the sequences with FPKM values of 0 were removed. At the same time, the contaminant sequences were removed according to the LCA-based taxonomy classification method. The N50 and GC content for each assembly were also calculated using NGS QC Toolkit (v2.3.3)^60^ **(**Table 3**)**.

We then employed BUSCO (v3)^61^ to evaluate the completeness of the final assembly using the 1,440 Embryophyta expected genes database (version 2). This analysis showed (Table 7) that 1,285 (89.24%) and 50 (3.47%) of the 1,440 expected Vertebrata genes were identified as complete and fragmented, respectively, while 105 (7.29%) genes were considered missing in the final assembly. BUSCO was also used to evaluate the completeness of the final gene set, and 87.99% of the 1,440 expected genes were identified (Table 7).

**Table 7:** Summary of BUSCO and PLAZA results.

Software	Gene type	Transcriptome		The final coding genes	
		Number	Percentage	Number	Percentage
BUSCO	Complete	1,285	89.24%	1267	87.99%
	Single-copy	937	65.07%	933	64.79%
	Duplicated	348	24.17%	334	23.19%
	Fragmented	50	3.47%	48	3.33%
	Missing	105	7.29%	125	8.68%
PLAZA CoreGF (GreenPlants)	Weighted score	98.85%		97.13%	
	Missing genes	33	1.13%	85	2.90%

To assess the completeness of the core gene families (CoreGFs) within the green plant lineage, the CoreGF score was calculated using PLAZA (v2.5)^62^. First, the assembled transcriptome sequences were searched against the CoreGF gene set using BLASTx, and the protein sequences of high-quality CDSs were searched against the CoreGFs using BLASTp (V2.4.0) with e-value < 1e-5. Then, scores were calculated using the script coreGF_plaza2.5_geneset.py in PLAZA based on the BLAST hits. The CoreGF weighted score was 98.85%, and only 33 out of 2,928 CoreGFs were missing from the final assembly, while the score was 97.13%, and only 85 CoreGFs were missing in the high-quality coding gene set.

### Contamination screening

**Fig. 5:**
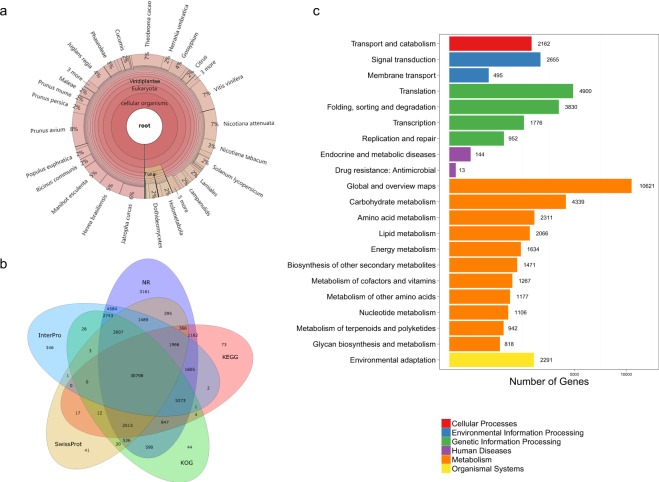
Functional annotation. (**a**) Venn diagram of annotations based on the databases NR, KOG, KEGG, Swiss-Prot and InterPro. (**b**) Species distribution of annotated database NR sequences. (**c**) KEGG pathway annotations. Bars represent the numbers of unigenes clustered into KEGG Orthology (KO) hierarchies.

The transcriptome sequences were subjected to contamination screening as mentioned in the methods, and the contaminant sequences were removed. The results of the contamination analysis also showed that (1) the main contaminants came from fungi or Arthropoda; (2) the most abundant fungi were *Paraphaeosphaeria sporulosa* in the Ascomycota; and (3) the transcriptome showed the highest similarity with *Prunus avium*, followed by *Theobroma cacao* and *Nicotiana attenuata*, in Viridiplantae (Fig. 5a).

sRNAs were assembled using velvet (v1.2.10)^63^ with the parameters “velveth Assemout 15-short -fastq.gz” and “velvetg Assemout”. Assembled contigs with lengths greater than 100 bp were extracted to detect potential symbionts using the virus detection pipeline published previously^64^. In brief, a total of 115 queries with lengths greater than 100 bp were searched against the NCBI virus database using BLASTn with e-value < 1e-5. All matched sequences were searched against the Nt database using BLASTn with e-value < 1e-5 to identify false positives. After detection, none of the known viruses were detected in the sRNA data. In addition, sRNA reads mapped to the contaminant transcriptome sequences were removed from further analysis.

### Annotation quality control

Statistical results of the functional annotation are summarized in Table 4 (Fig. 5b). A total of 65,534 (62.75%) unigenes were annotated. The distribution of KEGG pathway annotations is shown in Fig. 5c.

Statistical results for the predicted CDSs are summarized in Table 5. A total of 58,561 coding regions were detected by TransDecoder. The total, maximum and minimum lengths of the predicted CDSs were 60,633,393, 12,852 and 297. N50 and GC content were also calculated. Statistical results for the final high-quality coding genes are also summarized in Table 5.

## Data Availability

All analyses were performed using open source software tools, and the detailed parameters for each tool are shown in the relevant methods.
